# Intensity-Modulated Polymer Optical Fiber-Based Refractive Index Sensor: A Review

**DOI:** 10.3390/s22010081

**Published:** 2021-12-23

**Authors:** Chuanxin Teng, Rui Min, Jie Zheng, Shijie Deng, Maosen Li, Li Hou, Libo Yuan

**Affiliations:** 1 Guangxi Key Laboratory of Optoelectronic Information Processing, School of Optoelectronic Engineering, Guilin University of Electronic Technology, Guilin 541004, China; shijie.deng@guet.edu.cn (S.D.); lmss0830@163.com (M.L.); lbyuan@guet.edu.cn (L.Y.); 2 State Key Laboratory of Cognitive Neuroscience and Learning, Center for Cognition and Neuroergonomics, Beijing Normal University at Zhuhai, Zhuhai 519087, China; rumi@doctor.upv.es; 3 State Key Laboratory on Integrated Optoelectronics, College of Electronic Science and Engineering, Jilin University, Changchun 130012, China; zhengjie@jlu.edu.cn; 4 State Key Laboratory for the Chemistry and Molecular Engineering of Medicinal Resources, School of Chemistry and Pharmaceutical Science, Guangxi Normal University, Guilin 541004, China

**Keywords:** polymer optical fiber, refractive index sensing, intensity modulation, different structures

## Abstract

The simple and highly sensitive measurement of the refractive index (RI) of liquids is critical for designing the optical instruments and important in biochemical sensing applications. Intensity modulation-based polymer optical fiber (POF) RI sensors have a lot of advantages including low cost, easy fabrication and operation, good flexibility, and working in the visible wavelength. In this review, recent developments of the intensity modulation POF-based RI sensors are summarized. The materials of the POF and the working principle of intensity modulation are introduced briefly. Moreover, the RI sensing performance of POF sensors with different structures including tapered, bent, and side-polished structures, among others, are presented in detail. Finally, the sensing performance for different structures of POF-based RI sensors are compared and discussed.

## 1. Introduction

The refractive index (RI) is an important optical parameter of material. Some physical quantities such as concentration, temperature, and pressure, etc., can be reflected by the change of RI [1]. Therefore, RI measurement has great value of applications and is widely used in disease diagnosis [2], environmental monitoring [3], food safety [4], and biochemical sensing fields [5]. Optical fiber sensing technology employs the optical fiber to detect the optical information changes caused by the interaction between the transmitted light and the analytes. This technique first appeared in the 1960s, and with the development of optical fiber technology and optical fiber communication technology, the optical fiber sensing technology has gradually developed in recent decades. Compared with the electrical sensors, optical fiber sensors have the advantages of electromagnetic immunity, chemical corrosion resistance, electrical isolation, and are capable of distributed sensing and remote sensing, which makes them suitable for employing in the occasions when electrical sensors cannot be used [6].

To date, many different kinds of optical fiber-based RI sensors have been proposed. For example, the fiber grating-based sensors including the fiber Bragg gratings [7,8,9], the long-period fiber gratings [10,11,12], and the titled fiber Bragg gratings [13,14,15] were employed for RI sensing; different types of fiber interferometers, such as the Mach–Zehnder interferometer [16,17,18], the Fabry–Pérot interferometer [19], the Michelson interferometer [20], and the Sagnac interferometer [21,22], were used for RI measurement; many kinds of optical fiber-based surface plasmon resonance sensors were proposed for RI sensing [23,24,25]. Besides, some 2D materials like graphene [26] and molybdenum disulfide (MoS_2_) [27] were also integrated on the fiber for RI measurement. Most of the reported optical fiber RI sensors are based on the glass optical fibers; however, after the structural modification, they will become very fragile, which makes them unsuitable for RI measurement in some special situations. Compared with the glass optical fibers, the polymer optical fibers (POFs) can solve these issues. This kind of fiber is made of polymer materials, which have an ease of fabrication and operation, low cost, high flexibility, softness, and light weight [28,29]. The POF-based sensors have the advantages of high strain limit, ease of operating, and high repeatability, and can be used in harsh environments and bending situations [30,31]. In addition, polymer materials are more easily doped with organic materials [32] and compatible with biological materials, which gives POF-based sensors great potential in the biological sensing fields [33]. To date, the POF-based sensors have many applications including temperature detection [34], liquid level detection [35,36], displacement detection [37], PH detection [38], strain detection [39], bending measurement [40], environment detection [41], and so on.

According to the usage of optical fibers, optical fiber sensors can be classified into extrinsic or intrinsic sensors. For the extrinsic fiber sensor, the fiber is just acts as the input and output parts of the optical sensor, and does not participate in the modulation of the measured signals [42]. The intrinsic fiber sensors directly use optical fiber as the sensing material and the transmission medium, which carries the measured optical signal modulated in the optical fiber. The POF-based RI sensors reviewed in this paper belong to the intrinsic fiber sensors. On the other hand, according to the demodulation methods, the optical fiber sensors can be classified into intensity modulation, wavelength modulation, phase modulation, and polarization modulation. At present, POF-based RI sensors are mainly based on the wavelength modulation and intensity modulation methods. The POF-based SPR sensors [43,44,45,46], the POF grating-based RI sensors [47,48,49,50], and the POF interferometer-based RI sensors [51,52] are often working on the wavelength modulation mode. However, these types of RI sensors usually require a complicated fabrication processes, which is costly and time consuming, and the wavelength modulation method also needs expensive demodulation devices, which is also costly. Although the measurement accuracy of this type of sensor is high, the cost of its sensor systems is also high, which is not conducive to commercial applications. Compared with the wavelength modulation, the intensity modulation method does not need the complex fabrication processing and expensive equipment; usually, the cheap LED and photodetectors can be used as the light source and detector [53] for the sensor system. This type of sensor is very easy to implement and goes very well with multimode fiber, which could provide a low-cost solution for RI measurement. 

To our knowledge, there are only a few reviews that specifically address the optical fiber-based RI sensors. Recently, Xu et al. [54] introduced a wide range of most representative plasmonic and photonic sensors and placed them into a single map. Patil et al. [55] reviewed the optical fiber-based RI sensors and tried to make a comparative study of various existing devices and systems in this field. All of these existing reviews, however, are completely lacking information about intensity-modulated POF-based RI sensors. In this work, a brief review of the intensity-modulated POF-based RI sensors is presented. This paper does not aim to present a thorough review of all POF-based RI sensors, but rather to focus on more simple and low-cost interrogation approaches based on intensity variation measurement techniques. The background of RI measurement and the application of POF to RI measurement are briefly discussed in the first section of this paper. In the second section, the polymer fiber technology is summarized, and the working principle of the intensity-modulated POF-based RI sensors is introduced in the third section. In the fourth section, we introduce a variety of intensity-modulated POF-based RI sensors. Finally, a summary and outlook are provided.

## 2. Polymer Optical Fibers

The first POF was developed by DuPont in 1966, which appeared almost simultaneously with glass optical fiber [56]. However, compared with glass optical fibers, the POFs received much less attention. The POFs are not suitable for long-distance communication due to their high transmission loss. However, the advantages of POFs are that they are easy to handle, have good flexibility, and have a low loss window in the visible wavelength, which make them an ideal choice for short-distance communication and sensing applications. After many years of research and development, POFs made of different materials and different structures have been developed to improve their properties. The following sections mainly review the materials and structures of POFs.

### 2.1. Materials of POFs

**Figure 1:**
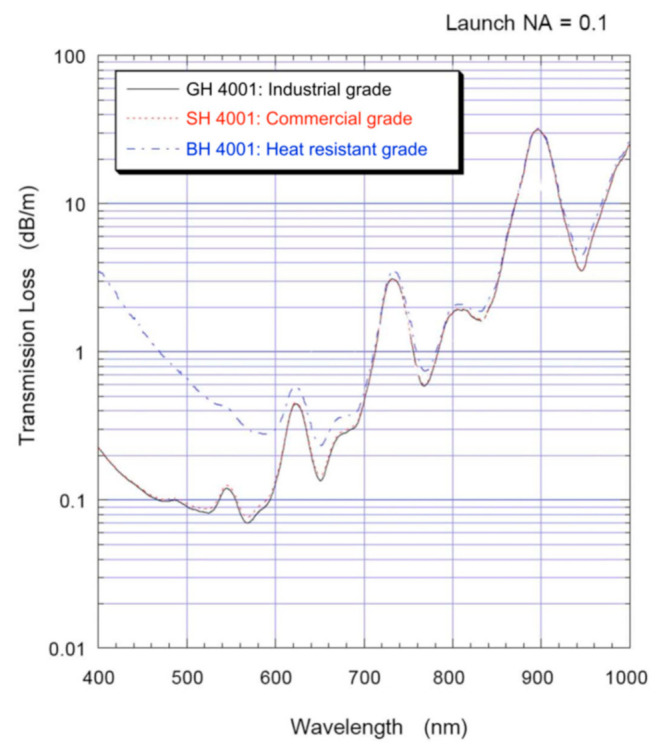
The typical transmission loss spectra of PMMA-based POFs from ESKA^TM^ [65].

A variety of materials have been used to fabricate POFs, including Polymethyl Methacrylate (PMMA) [57], polystyrene (PS) [58], polydimethylsiloxane (PDMS) [59], cycloolefin polymer (COC) [60], polycarbonate (PC) [61], perfluorinated polymer [62], silicone [63], and cycloolefin copolymer [64]. Different materials of POFs have different characteristics and applications. For example, PMMA has the low loss transmission characteristic in the visible region, which is the most commonly used commercial POF material. Figure 1 shows the typical transmission loss spectra of PMMA-based POFs from ESKA^TM^ [65]. Compared with the PMMA-based POFs, PC-based POFs have better heat resistance, which make them suitable to be used in a high-temperature environment. The perfluorinated polymer fibers (CYTOP) possess the feature of low material dispersion, which gives them a larger bandwidth.

### 2.2. Structures of POFs

**Figure 2:**
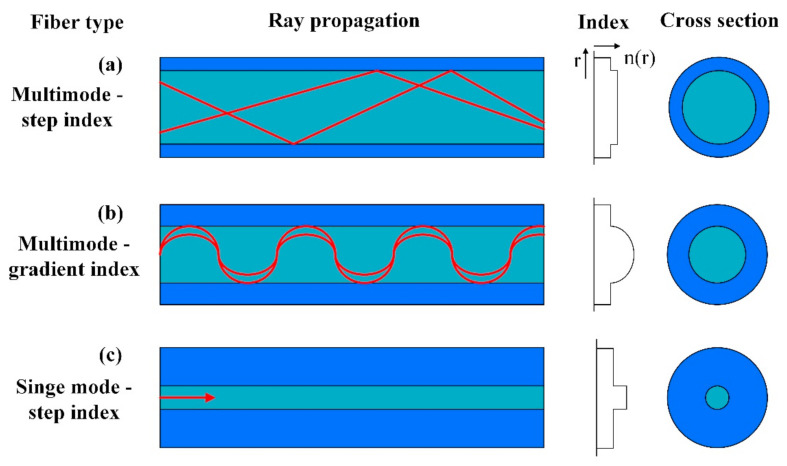
The common structures of POFs.

The most common and the first development structure of a POF is the step-index (SI) multimode POF. The schematic illustration of this structure is shown in Figure 2a. It consists of two layers: the core with a large diameter and the cladding with a small thickness. The common core material of this type of POF is PMMA with a larger RI, and the common cladding material of this type of POF is a fluorinated polymer with a lower RI [66]. This type of fiber can transmit a large number of modes, and it has been used in the sensing fields for a long time with the intensity demodulation method generally. Another common structure is the graded-index (GI) multimode POF, as shown in Figure 2b, the core material of this type of POF is often perfluorinated polymer with a graded RI [67]. Compared with the PMMA-based POFs, the GI perfluorinated polymer-based POFs have the low loss transmission characteristic at the communication wavelengths of 850 nm and 1300 nm. The diagram of SI single-mode POF is shown in Figure 2c [68]. This type of POF can propagate only one mode at a fixed wavelength, it has the significance applications in the development of POF-based gratings. Besides, the microstructured POFs including the photonics crystal fiber (PCF) [69] and the multicore fiber [70] have also been developed and proposed for sensing applications. 

## 3. Principle for Intensity-Modulated RI Sensing

For intensity modulation optical fiber-based RI sensors, the change of RI is measured by detecting the propagation loss of light, which is induced by refraction loss, evanescent field, optical absorption, etc. At the effect of total internal reflection, light is bounded inside the fiber core, and the critical angle $\theta_{c}$ at which total reflection occurs can be expressed as,

(1) $$\theta_{c}=sin^{-1}\left(n_{cl}/n_{co}\right)$$

where $n_{co}$ and $n_{cl}$ are the RIs of fiber core and cladding, respectively. For light propagated in fiber in the form of total internal reflection, some of the light power will go into the medium surrounding the fiber core, which is known as evanescent wave (EW). The energy of the EW decays exponentially at the direction perpendicular to the reflection interface. The penetration depth *d_p_* of EW can be expressed as,

(2) $$d_{p}=\frac{\lambda }{2\pi \sqrt{n_{co}^{2}sin^{2}\theta -n_{cl}^{2}}}\;$$

where *λ* is the wavelength of light in vacuum, and *θ* is the incident angle at the interface of core and cladding. In order to increase the sensitivity of RI sensor, the POF structure should be modified. Tapering, bending, and side polishing are the common processing method. For the tapered POF, the incident angle *θ*, and the cladding thickness will be decreased, and the decreased incident angle will lead to an increase of $d_{p}$, which could make the EW extend outside the cladding and react with the surrounding medium, and, hence, the output power will be changed with the RI variations of surrounding medium. For the bending POF structure, its principle is similar to that of tapered fiber. Besides, the fiber modes will shift to the outward direction, increasing the penetration depth as well. When the fiber cladding is removed, for example with the side-polished POF, the surrounding medium acts as the fiber cladding in the polished region; in this case, as the RI increases, the fiber will support less modes propagating in the fiber, and on the other hand, the penetration depth of EW will be also increased. Therefore, the RI changes of surrounding medium for these fiber structures can be detected by monitoring the output intensity.

## 4. Different Types of Intensity Modulation POF-Based RI Sensors

As mentioned above, the evanescent field intensity can be improved by modifying the fiber structure. The following presents the RI sensors based on POFs with different structures. 

### 4.1. Tapered POF-Based RI Sensors

**Figure 3:**
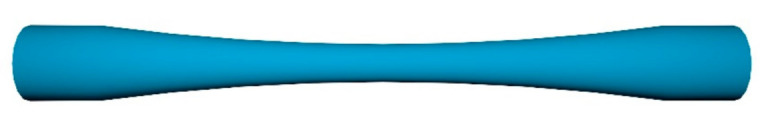
The schematic diagram of tapered POF.

The tapered structure is shown in Figure 3. It consists of a taper-decreasing region and a taper-increasing region. The transmission characteristics of light can be changed by this structure of fiber. In the decreased tapered region, the coupling and conversion of propagation modes of light are generated, and the total reflection condition of transmitted light is easily destroyed when the RI of surrounding environment changes, which will introduce the propagation loss. In addition, the tapering fiber diameter will cause a continuous change in the propagation angle, which will not only increase the number of total reflections in the fiber, but also bring more evanescent field power [71]. 

**Figure 4:**
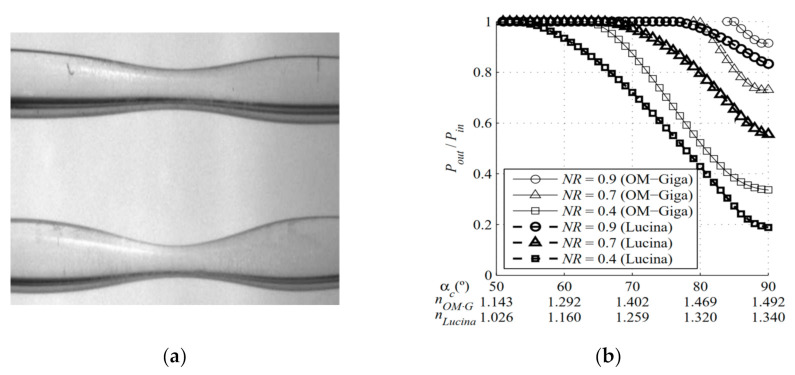
The photos of the tapered POFs (**a**), and the transmission behaviors of light for the OM-Giga/POF and the Lucina fibers with a different narrowing ratio in liquid with different RIs (**b**) [77].

The tapered POF can be fabricated by the heat-drawing method [72,73,74] or the chemical etching method [75,76]. Different heating sources including the furnace [72], the solder gun [52], and the flame [73] were used to heat the POF. Ujihara et al. [74] fabricated a tapered GI perfluorinated POF by a high-power light propagating inside the fiber, an approximately 4 mm long tapered region was obtained, and a RI sensitivity of 107 dB/RIU was achieved by using this fiber probe. In 2008, a tapered graded-index POF was fabricated and proposed for RI measurement by J. Arrue et al. [77]. They employed a ray-tracing method to analyze the behavior of light transmitted in the tapered GI POF and investigated the influence of the narrowing ratio on the RI sensitivity and RI measurable range for the OM-Giga/POF and the Lucina fibers as shown in Figure 4. They found that the narrowing ratio does not need to be very small for a large range of RIs to be measurable, but the maximum RI that can be measured is smaller than the case of the OM-Giga/Giga POF fibers. For the same narrowing ratio, the range of RIs that can be measured with a tapered Lucina POF is greater than that with a GI glass fiber.

Masayuki et al. [78] proposed a taper-type POF (with fiber core of PS material and cladding of PC material) sensor probe for ethanol solution measurement. The results showed that the proposed sensor can measure a low concentration of ethanol solution below 5 *v*/*v*%, whose sensitivity was about three times as that without the tapered structure. Besides, the sensor was temperature independent, and did not receive any influence from water, which was suitable for a real application. Yang et al. [71] optimized the tapered POF for the sensing of ethanol concentration. The ray-tracing method was used for theoretical investigation of different parameters of sensor, i.e., V-number matching, and evanescent wave penetration depth in this study. The theoretical analysis and experimental results were used to optimize the taper ratio and taper length for the achievement of high evanescent wave penetration depth and high sensitivity. The analysis indicated that the sensitivity of tapered fiber sensor can be improved by decreasing the taper ratio with simultaneous increase in the taper length. The highest sensitivity of 1.527 mV/% was achieved from the tapered fiber with a taper ratio of 0.27 and taper length of 8 cm. The proposed parametric optimized tapered fiber sensor can detect the change in concentration of C2H5OH as small as 6.55 × 10^−3^.

**Figure 5:**
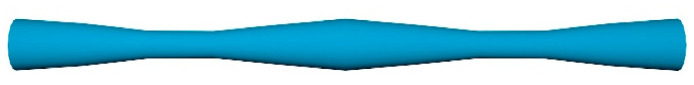
The schematic diagram of the double-tapered POF.

Rahman et al. [79] proposed and demonstrated a simple tapered POF sensor for continuous monitoring of salinity based on different concentration of sodium chloride (NaCl) in deionized water. The results showed that as the solution concentration varied from 0% to 12%, and the output voltage of the sensor increased linearly from 0.109 mV to 1.142 mV, with a sensitivity of 0.0024 mV/% and a linearity of more than 98%. Similar to this work, Feng et al. [73] proposed a RI sensor based on a taper POF. Three wavelengths (532, 633, and 780 nm) were used to evaluate the sensitivity of the sensor, and results indicated that 633 nm was the best sensing wavelength due to the increased levels of sensitivity achieved at this wavelength. Besides, a double-tapered fiber structure was designed to enhance the sensitivity of sensor as shown in Figure 5, and a sensitivity of 950 μW/RIU at 633 nm was obtained when the launched power was 1 mW.

Some nanomaterials, such as carbon nanotubes [80], graphene [81], and ZnO nanostructures [82], were coated on the tapered POF to enhance the RI sensing performance. Batumalay et al. [80] proposed a simple tapered POF coated with a single-wall, carbon nanotube, polyethylene oxide composite for the measurement of the uric acid concentration. The results showed that an improved sensitivity can be obtained from this sensor, as the solution concentration of the uric acid varied from 0 to 500 ppm, and the output voltage of the sensor had a linear response with a sensitivity of 0.0023 mV/% when the waist diameter was 0.45 mm and tapering length was 10 mm. Later, a graphene-coated tapered POF was proposed for uric acid detection by the same group [81]. The results showed that as the solution concentration of the uric acid varied from 0 ppm to 500 ppm, the output voltage of the sensor increased linearly with a sensitivity of 0.0021 mV/% and a linearity of more than 98.88%. Similar to these works, they employed a tapered POF coated with ZnO nanostructures for the measurement of different concentrations of uric acid in deionized water and the changes in relative humidity (RH) [82]. The results showed that as the concentration of the uric acid varied from 0 ppm to 500 ppm, and the output voltage of the sensor using tapered POF with seeded ZnO nanostructures increased linearly with a higher sensitivity of 0.0025 mV/ppm compared to 0.0009 mV/ppm for unseeded tapered POF coated with ZnO.

### 4.2. Bending POF-Based RI Sensors

**Figure 6:**
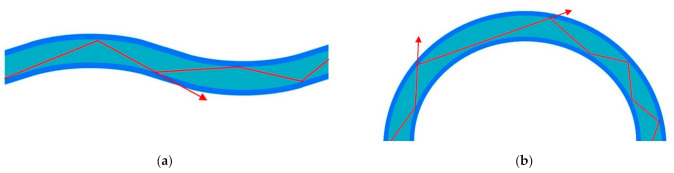
The schematic illustration of the micro-bending (**a**) and macro-bending POFs (**b**).

Similar with the tapering structure, bending the fiber can also increase the evanescent field power, which is mainly caused by the changes of RI profile and mode field distribution [83]. The bending can be classified into micro-bending and macro-bending. Micro-bending fiber usually means that the curvature radius of the fiber is comparable with the diameter of the fiber, as shown in Figure 6a. While for the macro-bending fiber, the curvature radius is usually much larger than the fiber diameter as shown in Figure 6b [84].

**Figure 7:**
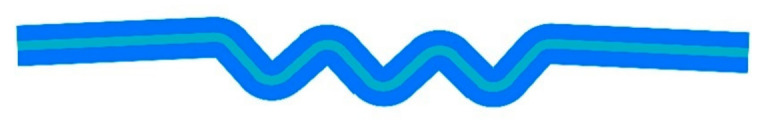
The schematic diagram of the permanent micro-bending POF.

Thomas et al. [85] proposed a permanently micro-bent bare POF for detecting chemical species as shown in Figure 7. The results showed that the output intensity is linearly dependent on the logarithm of concentration of the absorbing species surrounding the bent portion of the fiber, and the sensor can even detect very low concentrations in the order of nanomoles per liter with a dynamic range of greater than six orders of magnitude. George et al. [86] employed the similar fiber structure to detect the continuously varying RI of chlorinated water. The results showed that the evaporation of chlorine from water and the change in RI followed a first-order exponential decay function of time.

**Figure 8:**
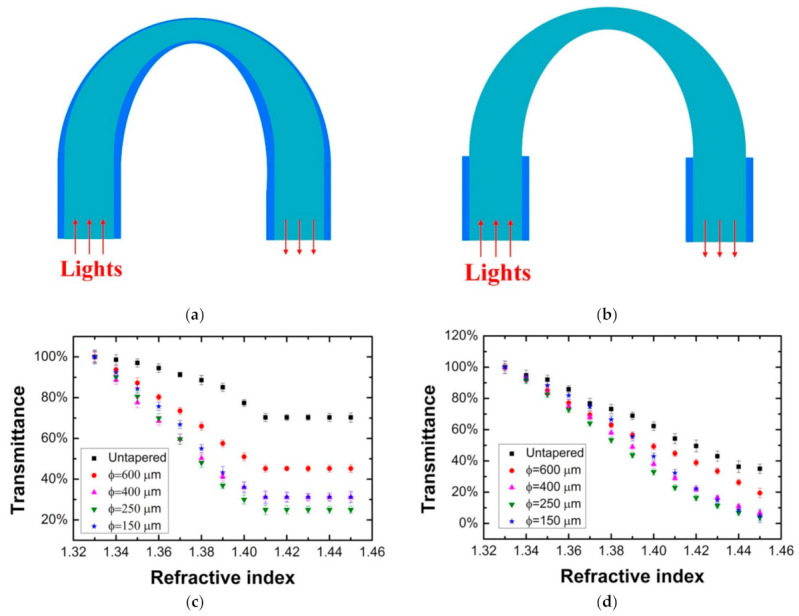
The schematic illustration of the macro-bending tapered POF probes with (**a**) and without (**b**) cladding, and the RI sensing performance for the probes with (**c**) and without (**d**) cladding [87].

The macro-bending structure was often combined with the taper or side-polished structure to increase the RI sensitivity of sensor. For example, Teng et al. [87] proposed a macro-bending tapered POF for the RI sensing as shown in Figure 8. The RI sensing performance for the probes with and without cladding was investigated. By changing the taper waist and curvature radius, the sensing performance of the probe was optimized. The highest sensitivity of the probe with cladding reached 937%/RIU in the RI range of 1.33–1.41, for the probe without cladding, the RI sensing range expanded to 1.33–1.45, and the sensitivity was about 800%/RIU. The temperature dependence of the probe with cladding was also investigated by the same group [88]. Wandermur et al. [89] manufactured a U-shaped probe with a specially developed device. The RI sensing performances for the probes with different structure parameters were compared. After functionalizing with antibody anti-*E. coli* serotype O55, the probe was tested with bacterial concentrations of 10^4^, 10^6^, and 10^8^ colony-forming units/mL (CFU/mL), and the decaying parameters of 3.0 × 10^−3^, 3.6 × 10^−3^, and 8.0 × 10^−3^ were obtained, respectively.

**Figure 9:**
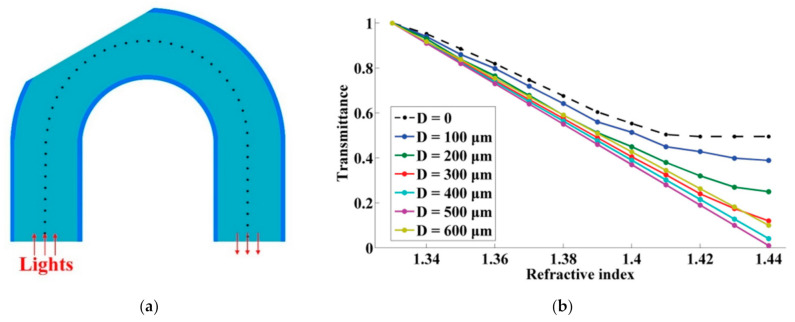
The schematic diagram of the side-polished macro-bending POF (**a**), and the effect of polished depth on the RI sensing performance (**b**) [90].

Jing et al. [90] proposed a side-polished, macro-bending POF for RI sensing as shown in Figure 9. By changing the curvature radius, the polished depth, and the polished position (angle), the RI sensing performance of the probe was optimized. They obtained the maximum RI sensitivity of 154 dB/RIU in the RI range of 1.33–1.44 when the curvature radius, the polished depth, and the polished position were 5 mm, 500 µm, and 60°, respectively. The influence of the temperature was also tested by the same group [91]. Wang et al. [92] demonstrated a U-shaped, double-sided, polished POF for RI sensing. They optimized the processing parameters experimentally, and a sensitivity of 1541%/RIU was obtained with a resolution of 5.35 × 10^−4^ in the scope of 1.33–1.39. Besides, Zhong et al. [93] explored the temperature-independent operation of a POF-based evanescent wave sensor immersed in distilled water. They observed that the light transmission modes and sensitivity of the sensor were affected by changes in the surface morphology, diameter, and RI of the sensing region caused by changes in temperature. The transmitted light intensity of the sensor was maintained at a constant level after five cycles of the heating–cooling treatment, after which the fibers exhibited a smooth surface, low RI, and large fiber diameter.

**Figure 10:**
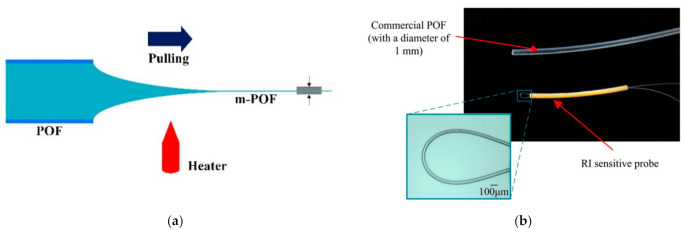
The schematic diagram of the directly drawing process of micro-POF from commercial POF (**a**), and the photo of the micro-POF (**b**) [94].

In addition, due to the large evanescent field of micro-fiber, the macro-bending micro-POFs were proposed for RI sensing [94,95,96,97]. Jing et al. [94,95] fabricated a micro-POF directly from the commercial POF and proposed a RI sensor based on a macro-bending micro-POF as shown in Figure 10. The macro-bending structure of the m-POFs was simulated and optimized by using the ray-tracing method. A linear RI sensing response was obtained with the sensitivity of 500%/RIU when the ratio of the radius of curvature of the macro-bending fiber to the radius of the fiber was 20. Irawati et al. [96,97] drew the micro-POF from melting PMMA and fabricated a micro-fiber loop resonator; after coating a layer of ZnO nanostructure, it was used for measuring the changes of relative humidity, with a variation from 20% to 80%. The experiment results showed that the output power of the sensor decreased linearly from −9.57 dBm to −20.19 dBm with a maximum sensitivity, linearity, and resolution of 0.1746 dBm/%, 94%, and 6.17%, respectively.

### 4.3. Polished POF-Based RI Sensors

**Figure 11:**
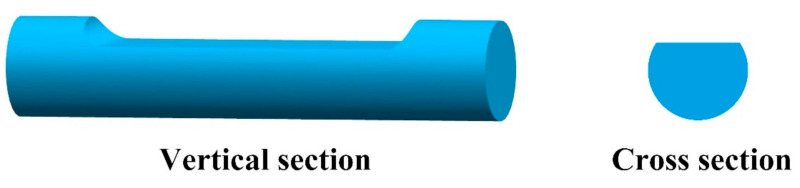
The schematic diagram of the side-polished POF.

The side-polished fiber removes parts of the fiber cladding or the core to increase the evanescent field power to interact with the surrounding medium. Because the shape of its cross section is similar to the English capital letter ”D”, it is also called the D-shaped optical fiber, as shown in Figure 11. 

Banerjee et al. [98] described the experimental results on RI sensing by a low-cost plastic-cladded POF and a silica fiber with a plastic coating that forms a protective layer on the silica cladding. The cladding of these two fibers were stripped to different thickness to make the fibers sensitive to RI of the environment. The results showed that the sensitivity of the sensor to RI change was nonlinear and was dependent on cladding thickness, and the sensitivity would reach a maximum at an intermediate thickness value. L. Bilro et al. [99] presented a cure monitoring system based on a side-polished interface on POF, and they also presented the modeling of a side-polished POF as a sensor for RI and curvature measurement by using a geometric optic approach [100]. The model considered different details such as the geometric description of the sensor, the intensity profile of the emitter, and the possibility of a multireflection for a light ray at the sensitive area. Teng et al. [101] investigated the RI sensing performances of the straight and macro-bending side-polished POFs. Results showed that the macro-bending probe had an enhanced RI sensing performance, and when the polished depth was 400 μm, the polished length was 10 mm and the curvature radius was 2 mm, and a sensitivity of 864%/RIU and a resolution of 3.3 × 10^−4^/RIU with a standard deviation of 0.16 were obtained.

Feng et al. [102] made a RI sensor based on a D-shaped POF, and different depths of the D-shaped groove and a different curvature radius of the fiber probe were used to research the influence for the sensor sensitivity. Experiment results showed that the proposed sensor had a good linear response for the measured RI ranging from 1.333 to 1.455, and the highest sensitivity of the sensor was obtained when the depth of the D-shaped fiber was 500 μm. They also simulated the energy distribution of the D-type structure by using the Finite Element Method [103], and the experiment results showed that the normalized transmittance intensity decreased 13.4% with the RI increasing from 1.333 to 1.455 when the depth and length were 500 µm and 2 cm, respectively, with an excurvature radius of 5 cm and optical source wavelength of 652 nm. 

In addition, Sequeira et al. [104] reported the optimization of the length for a D-shaped POF sensor for RI sensing from a numerical and experimental point of view. Results showed that, in the RI range of 1.33–1.39, the sensitivity and the resolution of the sensor were strongly dependent on the sensing region length, and the highest sensitivity resolution of 6.48 × 10^−3^ RIU was obtained with a 6 cm sensing length. While in the RI range of 1.41–1.47, the length of the sensing region was not a critical aspect to obtain the best resolution. Besides, Zhong et al. [105] investigated the mechanism of the effect of heat treatments on physical and optical properties of D-shape POF-based EW sensors.

### 4.4. Grating Structure-Based RI Sensors

The fiber gratings usually refer to the fiber Bragg gratings (FBG), the long-period gratings (LPG), and the titled Bragg gratings, which are fabricated on the single-mode silica fibers [106] or the single-mode polymer fibers [48]. They often work on the wavelength modulation method. While in this review, the grating structures introduced were fabricated on the multimode POF by a simple mechanical die press print method; due to the coupling of the core modes and cladding modes that occurs at all wavelengths, this type of fiber sensors work on the intensity modulation mode. 

**Figure 12:**
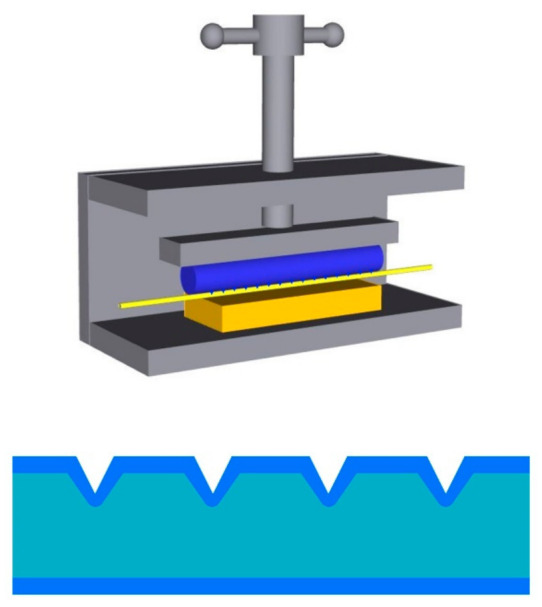
The schematic diagrams of the fabrication process of multi-notched structure on POF, and the structure of multi-notched POF [107].

In 2017, Teng et al. [107] proposed a POF with a multi-notched structure as a long-period grating for RI sensing. The structure was simply made on the surface of the fiber by pressing a thread rod against the POF as shown in Figure 12. The RI sensing performances for straight and macro-bending POFs with this structure were studied. Results showed that the POF probes with straight multi-notched structures were not sensitive enough for RI measurement. After bending the multi-notched structure into U-shaped probes, the RI sensing performance was improved markedly. The highest sensitivity of 1130%/RIU with a resolution of 8.44 × 10^−4^ RIU in the RI range of 1.333–1.410 was obtained. 

**Figure 13:**
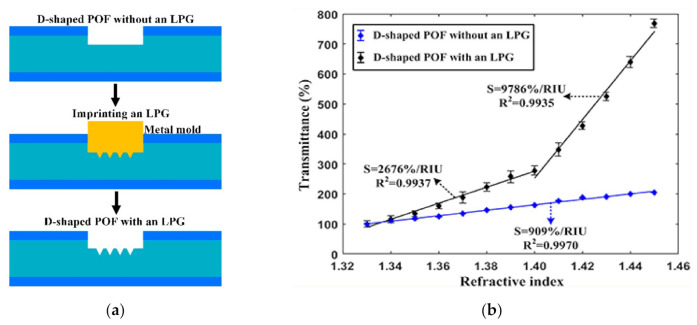
The schemes diagram of fabrication pross of LPG structure on POF (**a**), and (**b**) is the comparison of the experiment data for the D-shape POF probe with and without LPG structure [108].

In 2019, Xue et al. [108] fabricated the LPGs on the POFs with different diameters and investigated their RI sensing performances. The results showed that a higher RI sensitivity can be obtained when the LPG structure was imprinted on a thin POF (with a diameter of 0.25 mm), and the optimum sensitivity of 2815%/RIU with a resolution of 1.39 × 10^−4^ RIU was achieved in the RI range of 1.33–1.45 when the grating period, the groove depth, and the tilted angle were 100 µm, 65 µm, and 20 ^°^, respectively. Later, the same group proposed a D-shaped POF assisted by an LPG structure for RI sensing [109], as shown in Figure 13. The results showed that the LPG structure could achieve an enhanced RI sensitivity. When this structure was fabricated on POF with a thin diameter of 0.25 mm, the high sensitivities of 2676 %/RIU and 9786 %/RIU could be obtained in the RI ranges of 1.33–1.40 and 1.40–1.45, respectively.

**Figure 14:**
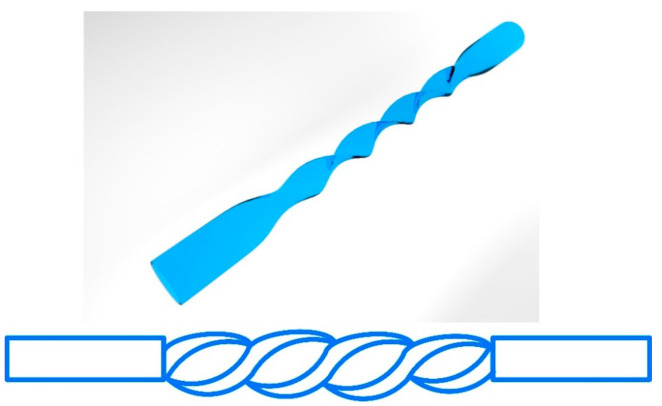
The schematic diagram of the screw-shaped POF [110].

In addition, a screw-shaped POF was fabricated through a heat pressing and twisting method and proposed for RI sensing by this group, as shown in Figure 14 [110]. This structure can lead to periodic coupling between core modes and cladding modes, which is similar to the working principle of LPG. The results showed that when the screw-shaped POF was fabricated by twisting a thin flat-shaped POF with a thickness of 600 µm and with a screw pitch of 2 mm, the highest sensitivities of 2277%/RIU, 4318%/RIU, and 4399%/RIU with the resolutions of 3.10 × 10^−4^ RIU, 1.63 × 10^−4^ RIU, and 1.60 × 10^−4^ RIU were obtained in the RI ranges of 1.33–1.37, 1.37–1.40, and 1.41–1.45, respectively.

### 4.5. Others

**Figure 15:**
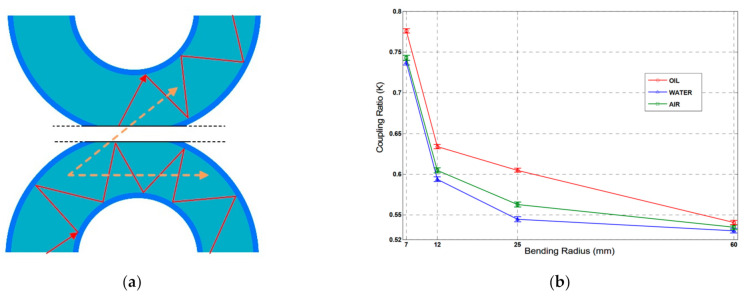
The schematic diagram of the side-polished POF coupler (**a**), and the variations of *K* for the probe with different bending radius in different liquids (**b**) [111].

Besides the above works, David et al. [111] proposed a self-referencing, fiber optic intensity sensor based on bending losses of a partially polished POF coupler, as shown in Figure 15. The coupling ratio (*K*) of the proposed sensor depended on the external liquid in which the sensor was immersed, which can be expressed as follows [111],

(3) $$T=\frac{4cos\theta {\left(cos^{2}\theta -cos^{2}\theta_{c}\right)}^{1/2}}{{\left[cos\theta +{\left(cos^{2}\theta -cos^{2}\theta_{c}\right)}^{1/2}\right]}^{2}}\;$$

where *θ* is the angle of incidence for a certain beam with the normal to the core surface and *θ_c_* is the critical angle. For *θ* ≤ *θ_c_*, the beam will be refracted from the fiber core, increasing the power losses. When the sensor was immersed into different liquids, these losses changed because of the different RIs surrounding the coupler, which could change the coupling ratio *K* of the sensor. The experimental results showed that the proposed sensor could distinguish and detect the presence of different liquids of the most usual liquids found in industry, like water and oil by the changes of *K*. Additionally, the coupling ratio *K* of the sensor had the increments of Δ*K* = 0.018 (from air to water), Δ*K* = 0.060 (from air to oil), and Δ*K* = 0.042 (from water to oil). Measurements also showed a low temperature dependence of *K*, below 1% from its nominal value. 

**Figure 16:**
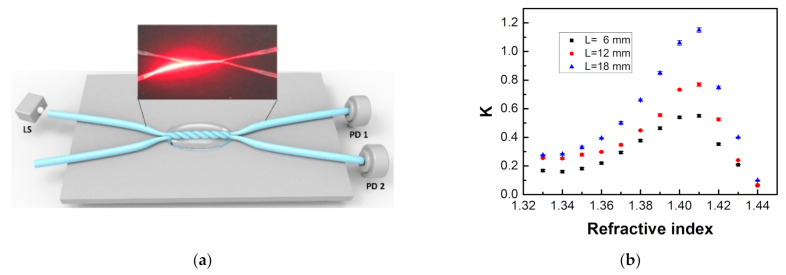
The schematic diagram of the twisted tapered POFs (**a**) and the RI sensing performances for the sensors with different twisted region length (**b**) [112].

Teng et al. [112] also proposed a similar coupling structure for RI sensing by employing two twisted tapered POFs. The tapered POFs were fabricated by a heating and drawing method and were twisted around each other to form a coupled structure. The sensor consisted of two input ports, a twisted region, and two output ports, as shown in Figure 16. The tapered POF could make the light couple from one POF to the other easily. When the RI of the external medium of the coupled region changes, the mode profile of the tapered POFs will be altered, leading to the changes in the coupling property. Therefore, the variations of the external medium RIs could be measured by monitoring the changes of the coupling ratio. Experiment results showed that when the active fiber diameter was 100 µm, the passive fiber diameter was 200 µm, and the twisted region length was 18 mm, the sensitivity reached 1700%/RIU and −3496%/RIU for the RI ranges of 1.37–1.41 and 1.41–1.44, respectively.

**Figure 17:**
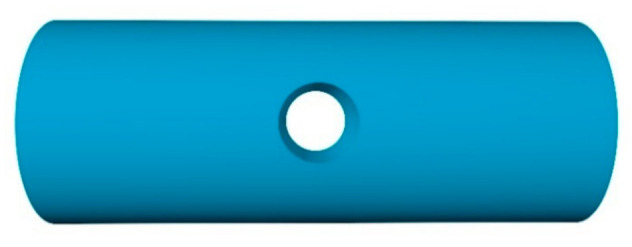
The schematic diagram of the side-hole structure POFs.

The side-hole structure fabricated on the POF was also investigated and proposed for RI sensing [113,114,115,116,117], as shown in Figure 17. Xin et al. [113] drilled a micro-hole by using a miniature numerical control machine for RI sensing in 2013. When the measured RI in the hole changes, the transmission behavior of the light will be changed accordingly. The experiment results showed that the sensor had a good linear relationship between the transmission and RI over a large operating range from 1.335 to 1.475, and a sensitivity of 36,071.43 mV/RIU (RI unit) was archived. The relationship between the transmission and the RI of the hole depended on the micro-hole’s diameter and depth. Later, they fabricated the micro-hole on the POF by using the femtosecond laser [114]. The experimental results showed that, in the RI operation range of 1.333–1.473, the sensor had a good linear loss (dB) response to the liquid RI in the micro-holes and a high RI sensitivity of 18 dB/RIU approximately. Shin et al. [116,117] proposed a RI senser probe with the similar structure, and a simple ray optics model was used to analyze the sensor transmittance with different liquids, and the difference between experimental and calculated results proved to be less than 6%.

**Figure 18:**
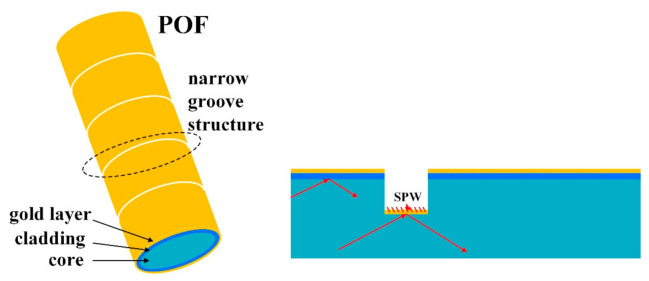
The schematic diagram of the narrow grooves structure, POF-based surface plasmon resonance sensor [118].

In addition, Hu et al. [118] coated a layer of gold film on the POF with narrow grooves structure to form the surface plasmon resonance sensor as shown in Figure 18. The proposed sensor was characterized using the intensity interrogation, where the change in transmission power was induced by light-filed interaction. Narrow groove structures with lengths of 5 mm were fabricated using an ultraviolet laser, and a gold layer was sputtered to the surface of the whole fiber. The experiment results showed that the sensor had a liner RI sensing response between 1.340 and 1.356. The highest sensitivity could reach 12.5 dB/RIU (126 µW/RIU) when the machining pitch was 400 µm.

## 5. Comparison of Intensity Modulation POF-Based RI Sensors

Table 1 shows the review of sensing performances, in terms of RI sensing range sensitivity, and resolution for various structures of POF-based RI sensors. After comparing the results from Table 1, some useful considerations can be concluded. The intensity modulation POF-based RI sensors can measure the RI in the range from 1.333 (water) to the RI closing to fiber core (or cladding). Although the units of sensitivity given in Table 1 are different, it can be also derived that the RI sensitivities are different for POFs with different structures, and the sensitivity of this type of sensor can satisfy the RI measurement in general applications. It can be also seen that the POF probe with an LPG structure possesses a higher sensitivity. On the other hand, the limitation of this type of sensor should be pointed; that is, their measurement accuracy is not very high, because the intensity modulation method is easily affected by light source fluctuation, detector noise, and environmental disturbance. So, in order to improve their performance, the more stable light sources and detectors are recommended, and it is necessary to choose the appropriate devices to build the sensor system according to the requirements of the applications.

## 6. Conclusions

In this review, the POF-based RI sensors working in intensity modulation were summarized. The properties of POF were introduced briefly, and a general description of the operation principles of the evanescent wave was presented as well. Several configurations of intensity modulation POF-based RI sensors were discussed, including the tapered POF, the bending POF, the side-polished POF, the side-hole POF, the POF with LPF structure, and the coupling structure POF. Different POF structure-based RI sensors show different sensing performances, and these kinds of sensors can achieve the satisfactory sensing range and sensitivity by optimizing the structure parameters. The relative simplicity, ease of implementation, and low cost are their main advantages, and by employing the low-cost light source and detector, it is easy to realize the intensity modulation fiber RI sensing system. However, most of the measured RI involved in this review refers to the bulk RI, which cannot meet the requirements in practical applications. To measure the RI changes of a specific substance, the additional bio-function modifications should be implemented on the fiber, which will have a potential application value in biochemical sensing fields.

## Figures and Tables

**Table 1:** Review of various structures of POF-based RI sensors.

Sensor Structure	RI Measurement Range	Sensitivity	Detection Limit or the Resolution	Ref.
Tapered POF	10–50 ethanol concentration	1.527 mV/%	6.55 × 10^−3^	[71]
Double-tapered POF	1.33–1.41	950 μW/RIU	-	[73]
Tapered POF	1.333–1.410	107 dB/RIU	-	[75]
Tapered POF	0–12% NaCl solution concentration	0.0024 mV/%	-	[79]
Tapered POF coated with carbon nanotubes	0–500 ppm uric acid	0.0023 mV/%	6.95 ppm	[80]
Macro-bending, tapered POF	1.33–1.41	937%/RIU	2.22 × 10^−3^	[87]
Side-polished, macro-bending POF	1.33–1.44	154 dB/RIU	-	[90]
U-shaped, double-sided, polished POF	1.33–1.39	1541%/RIU	5.35 × 10^−4^	[92]
Macro-bending micro-POF	1.33–1.45	500%/RIU	-	[94]
Side-polished POF	1.33–1.48	-	-	[100]
Macro-bending, side-polished POF	1.33–1.44	864%/RIU	3.3 × 10^−4^	[101]
D-shaped POF	1.333–1.455	-	-	[102]
U-shaped, multi-notched POF	1.333–1.410	1130%/RIU	8.44 × 10^−4^	[107]
Thin POF with LPG structure	1.33–1.45	2815%/RIU	1.39 × 10^−4^	[108]
D-shaped POF with LPG structure	1.33–1.45	2676 %/RIU (1.33–1.40) 9786 %/RIU (1.40–1.45) 2277%/RIU (1.33–1.37)	4.17 × 10^−5^ 1.14 × 10^−5^ 3.10 × 10^−4^	[109]
Screw-shaped POF	1.33–1.45	4318%/RIU (1.37–1.40) 4399%/RIU (1.41–1.45)	1.63 × 10^−4^ 1.60 × 10^−4^	[110]
Twisted tapered POFs	1.37–1.44	1700%/RIU (1.37–1.41) −3496%/RIU (1.41–1.44)	-	[112]
Side-hole structure POF	1.335–1.475	36,071.43 mV/RIU	-	[113]
Side-hole structure POF	1.333–1.473	18 dB/RIU	-	[114]
Narrow groove POF coated with gold film	1.340–1.356	12.5 dB/RIU (126 µW/RIU)	-	[118]
